# High Abundance of *Haemoproteus* Parasites in *Culicoides* (Diptera, Ceratopogonidae), with a Confirmation of *Culicoides reconditus* as a New Vector of These Avian Blood Parasites

**DOI:** 10.3390/insects15030157

**Published:** 2024-02-26

**Authors:** Carolina Romeiro Fernandes Chagas, Mélanie Duc, Margarita Kazak, Kristina Valavičiūtė-Pocienė, Dovilė Bukauskaitė, Carolina Hernández-Lara, Rasa Bernotienė

**Affiliations:** Nature Research Centre, Akademijos st. 2, 08412 Vilnius, Lithuania; melanie.duc@gamtc.lt (M.D.); margarita.kazak@gamtc.lt (M.K.); kristina.valaviciute@gamtc.lt (K.V.-P.); dovile.bukauskaite@gamtc.lt (D.B.); carolinahl85@gmail.com (C.H.-L.); rasa.bernotiene@gamtc.lt (R.B.)

**Keywords:** *Culicoides*, *Haemoproteus*, cytochrome *b*, sporozoite, microscopy, salivary gland, vector competence, Lithuania, sporogonic development

## Abstract

**Simple Summary:**

*Haemoproteus* parasites are one of the most studied avian blood parasites; however, their natural vectors, *Culicoides* biting midges, have been identified for only a small portion of them. The main reason for that might be due to the existence of a few research groups working with an integrative approach that allows not only the identification of parasite DNA in the insects, but also confirms the presence of the parasite infective stage (sporozoites) using microscopy. In this study, we aimed to identify the natural vectors of *Haemoproteus* parasites and to determine their prevalence in *Culicoides* biting midges in four different localities in Lithuania. Almost 2000 parous *Culicoides* females belonging to 11 species previously registered in the country were processed. The most abundant one was *C. pictipennis*. Parasite DNA was found in 7.9% of the analyzed biting midges, and sporozoites were present in almost 30% of these insects. The Botanical Garden was the locality with the highest abundance of biting midges and parasite species, as well as the place with the highest number of biting midges containing sporozoites. One new *Haemoproteus* vector (*C. reconditus*) was confirmed, and 12 new interactions between *Haemoproteus* parasite lineages and *Culicoides* biting midges of different species were identified. This study helps to understand the relationship between *Haemoproteus* parasites and *Culicoides* biting midges in the wild.

**Abstract:**

*Haemoproteus* parasites are the most diverse among Haemosporida. However, their natural vectors (*Culicoides*) are still poorly investigated and were identified for only a few parasite species and lineages. The application of an integrative approach (insect dissection, microscopic analysis, and molecular-based methods) is necessary in these studies, which have been carried out by a few research groups, mainly in Europe. The aim of this study was (i) to determine the *Culicoides* species that are naturally infected by *Haemoproteus* parasites, and which can support its complete sporogonic development, and (ii) to investigate the prevalence of *Culicoides* species and *Haemoproteus* parasite lineages in different study sites. In total, 1953 parous *Culicoides* females, from 11 species, were collected in four different localities in Lithuania and were dissected and analyzed using an integrative approach. The most abundant was *C. pictipennis* (30.3%). Parasite DNA was found in 7.9% of all investigated *Culicoides*, of which ~30% had sporozoites in their salivary glands, confirming their vector competence for these parasites. The Botanical Garden presented the highest number of *Culicoides* parous females, *Culicoides* species, and parasite lineages, as well as the highest positivity for sporozoites. *Culicoides reconditus* was confirmed as a natural vector of *Haemoproteus* parasites, sporozoites of six *Haemoproteus* lineages were reported for the first time, and 12 new interactions between *Haemoproteus* parasite lineages and *Culicoides* species were identified. *Haemoproteus* parasites seem to be transmitted by a high number of *Culicoides* species, with *C. kibunensis, C. pictipennis*, and *C. segnis* being the most important vectors.

## 1. Introduction

The genus *Haemoproteus* is the most diverse among the order Haemosporida, with *Culicoides* transmitting *Haemoproteus (Parahaemoproteus)* spp. and Hippoboscidae flies transmitting *Haemoproteus (Haemoproteus)* spp. [1,2,3,4,5]. This high parasite diversity is confirmed by the 177 species described [2], and the 2019 genetic lineages (the haplotypes of a fragment of the *cytochrome b* gene (*cytb*) of the parasite) reported in the MalAvi database (http://130.235.244.92/Malavi/, accessed on 20 November 2023) [6]. However, studies addressing the capacity of naturally infected *Culicoides* species to support the sporogony of *Haemoproteus* parasite (evidenced by the presence of sporozoites in salivary glands [7]) remain scarce. In fact, the lack of information regarding the natural vectors of other Haemosporida parasites (*Plasmodium* and *Leucocytozoon*) is very similar [8,9]. The main reason being the necessity to use an integrative approach, which consists of a combination of classical parasitology (with insect dissection and microscopic examination to confirm the presence of sporozoites) with molecular methods (to obtain the parasite DNA and confirm its species and lineage) [9]. Until now, such an approach was only used by a few research groups in the haemosporidian field, mainly in Europe [8,10,11,12,13,14]. 

Currently, only nine *Haemoproteus* species (5.1% of the described ones) and 11 lineages (0.5% of the described ones) have had their natural vectors identified [10,11,12,13,14]. This represents a small proportion of the existing parasite species and lineages and shows the huge gap that needs to be filled in the *Haemoproteus–Culicoides* system. 

In wild-caught and naturally infected *Culicoides*, from the 1347 described existing species [15], only five were confirmed to support the sporogonic development of *Haemoproteus*, all of them in Europe, namely *C. pictipennis*, *C. festivipennis*, *C. segnis*, *C. kibunensis*, and *C. punctatus* [10,11,12,13,14]. Previous studies have experimentally addressed this issue and showed that 11 other *Culicoides* species can support the sporogonic development of *Haemoproteus* [16]. However, many of these experimentally infected *Culicoides* are considered to be mainly mammalophilic [17,18,19,20], which means that their role as natural vectors of *Haemoproteus* parasites is limited and there is a small chance of the parasite to be transmitted to the next avian host by them, even though they occasionally feed on birds [18,19,21,22,23,24,25].

A recent study has pointed out a bigger number of interactions between certain *Culicoides* species and *Haemoproteus* lineages [11], e.g., *C. circumscriptus* has been more frequently reported to be PCR-positive to *Haemoproteus* lineages found in Accipitridae (hawks), Corvidae (crows and ravens), and Strigidae (owls) [26,27,28,29]. This might be explained by the fact that these vectors and bird species live closely to each other, at 20–26 m above the ground [30]. Similarly, *C. kibunensis* was shown to have a high number of interactions with parasite lineages that are commonly found in Turdidae (thrushes) birds, which live mainly at ground level [31]. This can also indicate that the density of these insects can differ at different heights [32,33], but as with many other topics related to these tiny insects, there is insufficient information.

That said, the objectives of this study are (i) to determine *Culicoides* species that are naturally infected by *Haemoproteus*, and which can support its complete sporogonic development, and (ii) to investigate the abundance of *Culicoides* species and *Haemoproteus* lineages in different study sites.

## 2. Materials and Methods

### 2.1. Collection of Biting Midges

**Figure 1:**
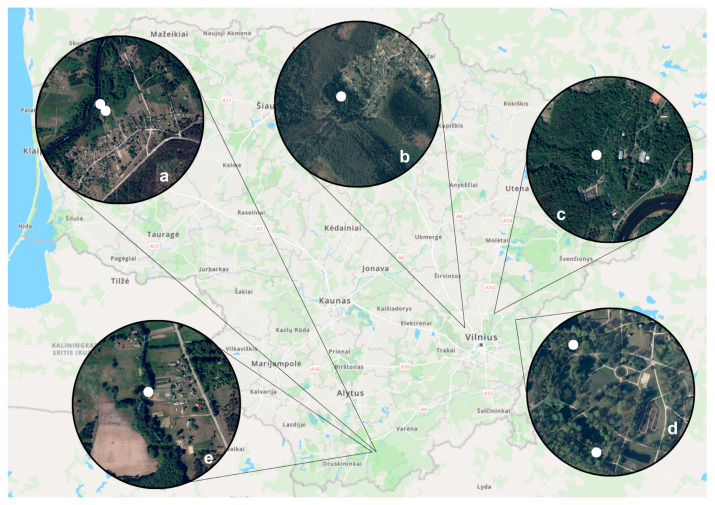
Study sites in Lithuania and trapping location marked with a white spot. Puvočiai village (**a**,**e**), Brinkiškės village (**b**), Verkiai Regional Park (**c**), and Vilnius University Botanical Garden (**d**).

Based on soil humidity, the presence of waterbodies, organically rich substrates, closed woods, and lack of wind, four trapping localities were selected for this study in Lithuania. They were the Vilnius University Botanical Garden, henceforward the Botanical Garden, (54°44′12.5″ N 25°24′16.4″ E), Puvočiai (54°06′52.2″ N 24°18′17.6″ E) and Brinkiškės (54°79′88.4″ N 25°06′03.7″ E) villages, and the Verkiai Regional Park (54°45′00″ N, 25°17′00″ E) (Figure 1).

In the Botanical Garden, trapping sites were chosen based on the presence of water bodies, small areas with relatively close deciduous woods, and the presence of blood-sucking insects that were indicated by the staff (Figure 1d). Meanwhile, Puvočiai village trapping sites included pine tree forest areas; domestic chicken and sheep farming areas; a trapping spot near the Merkys river; an artificial dug water pond for firefighting; and a spot near a camping site (Figure 1a,e). In Brinkiškės village, biting midges were collected near an artificially dug ditch that has been silted up over several years (Figure 1b). Finally, at the Verkiai Regional Park, located in Vilnius city limits, the traps were set close to swampy natural puddles (Figure 1c).

*Culicoides* were collected between June and September 2022, UV light traps (BG-Pro All-In-One Biogents AG) were placed between 1.5 and 2 m from the ground. The trapping effort and period varied for each location. Biting midges were collected in the Botanical Garden between the middle of June and the middle of September (seven nights); in Puvočiai village in the beginning of June (three nights); in Brinkiškės village between the middle of June and the middle of July (two nights); and in the Verkiai Regional Park between the beginning of June and the middle of July (three nights). 

**Figure 2:**
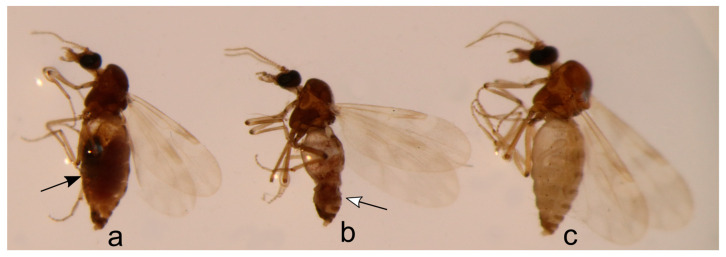
*Culicoides pictipennis* females: engorged (**a**), parous (**b**), and nulliparous (**c**). White arrow shows the burgundy pigment in the abdomen indicating that at least one gonotrophic cycle occurred (**b**). Black arrow shows blood meal in the abdomen (**a**).

Trapping was carried out between 6 and 7 h before sunset and between 4 and 5 h after sunrise. *Culicoides* were collected into a specimen jar filled with water and a drop of liquid soap (to break the water’s natural surface tension) and transported to the laboratory on the same day for processing. Using a binocular stereomicroscope, parous *Culicoides* females with burgundy pigment (Figure 2b) in their abdomen were separated and dissected (procedure is explained below). The presence of the burgundy pigment indicates that at least one gonotrophic cycle occurred, which means that they had at least one blood meal–for the majority of species–and increases the chances to yield *Haemoproteus* sporozoites [34].

### 2.2. Biting Midges Dissection, Identification, and Microscopic Examination of Salivary Gland Preparations

The dissection procedure consisted of placing the insect in a drop of 0.9% saline solution on a glass slide, removing the head and wings, transferring them to a new glass slide into a drop of Euparal, and covering them with a cover slide. These permanent preparations were dried in an incubator at 60 °C for one week and were used for the morphological identification of *Culicoides*, based on the available literature [35,36,37]. Salivary glands preparations were done by gently crushing the *Culicoides* thorax, preparing a small thin smear. To avoid DNA contamination, needles used for dissection were disinfected in fire after each dissection. These preparations were air-dried at room temperature, fixed with absolute methanol, and stained for 1 h with a 4% Giemsa solution [7]. Remnants of dissected *Culicoides* were transferred to a tube containing 96% ethanol and used for further molecular (PCR-based) analysis which is described below.

Only the salivary gland preparations of PCR-positive insects were microscopically examined. This was carried out using an Olympus BX-43 light microscope equipped (Olympus, Tokyo, Japan) with an Olympus DP12 digital camera and the image software Olympus DP-SOFT v.3.2 (Olympus, Tokyo, Japan). Each salivary gland preparation was entirely examined at high magnification (1000×). Representative preparations of sporozoites (NS49742-NS49757) were deposited at the Nature Research Centre, Vilnius, Lithuania.

### 2.3. DNA Extraction, PCR, and Sequencing Analysis

The insect remnants were used for the total DNA extraction, following an ammonium acetate extraction protocol [38]. *Haemoproteus/Plasmodium* DNA was amplified using a nested PCR protocol that amplifies a fragment of ~480 bp of the *cytb* gene [39,40]. One negative (nuclease-free water) and one positive (a sample with a single infection of *Plasmodium relictum cytb* lineage GRW4) control was included in each run. Successful DNA amplification was evaluated by electrophoresis using 2 μL of PCR product in a 2% agarose gel. Samples considered positive were the ones presenting a band with the same size as the targeted fragment. All PCR-positive samples were precipitated with ammonium acetate [38] and sequenced in both directions with corresponding primers using a Big Dye Terminator V3.1 Cycle Sequencing Kit and ABI PRISMTM 3100 capillary sequencing robot (Applied Biosystems, Foster City, CA, USA). 

Geneious Prime 2023.2.1 (https://www.geneious.com) was used to analyze the obtained electropherograms of sequences and to create a contig sequence. The presence of two or more peaks in the same position was considered as a mixed infection. Lastly, the contigs were compared with other sequences from the MalAvi database [6] using BLAST (Basic Local Alignment Search Tool). Lineage identities were considered when the obtained contig had 100% similarity with existing lineages. If at least one base pair of difference was seen in obtained sequences, they were considered as new lineages and named following MalAvi guidelines [6]. All obtained sequences were deposited in both GenBank (PP003069-PP003214) and MalAvi databases.

The morphological identification of *Culicoides* species was confirmed by the amplification of a ~680 bp fragment of the *cytochrome c oxidase subunit I* (*COI*) of the mitochondrial DNA of insects [41]. The PCR products obtained were sequenced from the 3′ end. Electropherogram analyses were conducted as mentioned above for the parasite DNA sequences and compared with other deposited sequences in the GenBank database using BLAST. Identifications were considered only if the sequence presented a similarity of ≥99%. The morphological identification was consistent with the PCR-based ones and all the sequences were deposited in GenBank (accession numbers OR995548-OR995567).

### 2.4. Statistical and Diversity Analyses

The determination of the prevalence of *Culicoides* species on each study site, PCR positive, microscopy positive samples, and parasite lineages were calculated using IBM SPSS Statistical v29.0.1.0 [42]. Due to differences in our sampling effort, rarefaction curve analyses were conducted with R v4.3.2 [43] using the *PAST* v4.16 package [44] with 95% confidence intervals (CIs), and estimated sample coverage (R package *SpadeR* v0.1.1, [45]) was calculated to determine if diversity indices could be compared among sites. Estimated sample coverage (C) is an objective measure of sample completeness [45]. 

In order to characterize the degree of heterogeneity among species incidence or detection probabilities, we estimated the coefficient of variation (CV) with R v4.3.2 [43] package *SpadeR* v0.1.1, [45]. If the CV is zero, then all the species in an assemblage have the same probability to be detected. On the other hand, when the CV increases, it indicates that the probability of species to be detected varies between species. 

Species richness (S_est_) along with their 84% confidence intervals (equivalent to statistical tests at α < 0.05 level [46]) were estimated with R v4.3.2 [43] package *iNEXT* v3.0.0 [47]. Therefore, species richness among assemblages is significantly different when their 84% CIs do not overlap. The exponential of Shannon index (e^H’^) was estimated for each site with the *SpadeR* v0.1.1 package [45]. A higher e^H^’ indicates a more evenly distributed abundance of species present in that community. Similarity in species composition between sites was assessed with a cluster analysis using the Bray–Curtis index with 100 bootstrap replications in *PAST* v4.16 [44]. 

## 3. Results

During the study, 1953 parous *Culicoides* females were collected and dissected. They belong to 11 different species, the most abundant ones were *Culicoides pictipennis* (30.3%), *Culicoides obsoletus* complex (23.7%), and *Culicoides kibunensis* (23.1%). The least common species were *Culicoides reconditus*, *Culicoides pulicaris,* and *Culicoides circumscriptus*, with five, three, and one individual, respectively. A few insects (five in total) could not be identified using either morphology or molecular analysis, and they are referred to as *Culicoides* sp. (Table 1).

**Figure 3:**
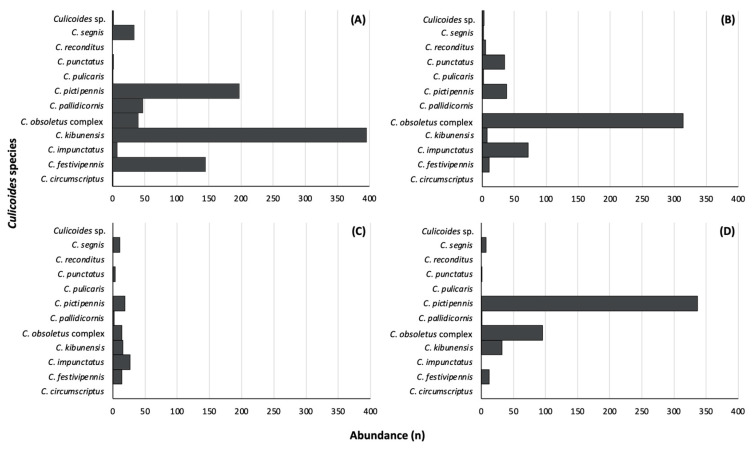
*Culicoides* species abundance (n) collected and analyzed from the Botanical Garden (**A**), Puvočiai village (**B**), Brinkiškės village (**C**), and the Verkiai Regional Park (**D**).

Concerning study sites, the majority of insects were collected at the Botanical Garden (871), followed by Puvočiai village (490), the Verkiai Regional Park (485), and Brinkiškės village (107). The highest number of *Culicoides* species was detected at the Botanical Garden (ten species), while the Verkiai Regional Park (seven species) was the location with the lowest number of species (Figure 3). At the Botanical Garden, the most abundant species was *C. kibunensis* (Figure 3A); while in Puvočiai village, biting midges from the *C. obsoletus* complex were more common (Figure 3B); and in the Verkiai Regional Park, *C. pictipennis* was more frequently found (Figure 3D). *Culicoides circumscriptus* was collected only at the Botanical Garden and *C. reconditus* was collected only in Puvočiai village. All biting midge species were found in low numbers in Brinkiškės village, with *C. impunctatus* being the most abundant one (Figure 3C).

**Figure 4:**
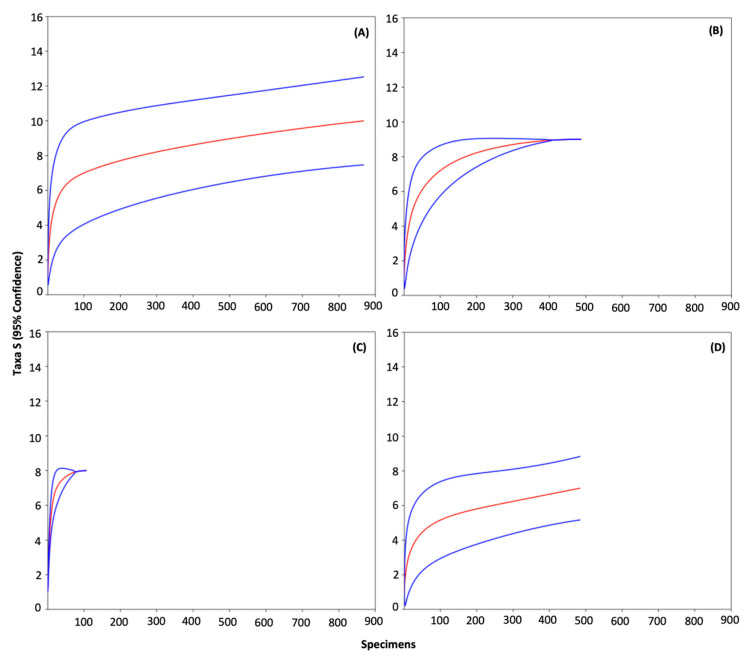
Expected *Culicoides* species richness rarefaction curves (red lines) with their 95% confidence intervals (CIs) (blue lines) in the Botanical Garden (**A**), Puvočiai (**B**) and Brinkiškės (**C**) villages, and the Verkiai Regional Park (**D**). Species are well represented with a specific sampling effort when the species richness rarefaction curve reaches an asymptote (i.e., (**B**,**C**)).

Rarefaction curves indicate that the asymptote was reached in Puvočiai (Figure 4B) and Brinkiškės (Figure 4C) villages, thus it seems that *Culicoides* species in those assemblages are well represented and new species are unlikely to be found by increasing the sampling effort (Table 2). Curves from the Botanical Garden (Figure 4A) and the Verkiai Regional Park (Figure 4D) present a weak slope but have not reached the asymptote. Therefore, it is possible that a few rare species could be found if sampling effort would be increased. However, the estimated sample coverage (C) from the Botanical Garden and the Verkiai Regional Park are almost one (Table 2), indicating that the sample completeness at those sites is very high. 

According to the coefficient of variation (CV), the probability of the detection of *Culicoides* species in the Puvočiai village is the least heterogenous from studied locations, followed by the Verkiai Regional Park, the Botanical Garden, and Brinkiškės village (Table 2). The Shannon diversity (exponential of Shannon entropy, e^H’^) of *Culicoides* species was significantly different among all sampling sites. The highest Shannon diversity was found at Brinkiškės village and the lowest at the Verkiai Regional Park (Table 2). In other words, abundances of *Culicoides* species were more equally distributed in Brinkiškės village than in any other site. On the contrary, the abundances of *Culicoides* species in Verkiai Regional Park were the most contrasting of our sampling sites.

**Figure 5:**
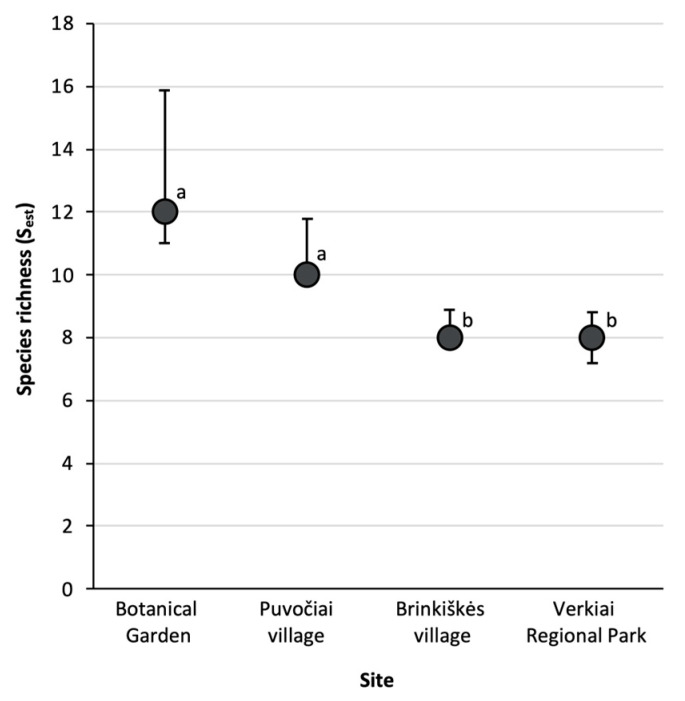
Estimated *Culicoides* species richness (S_est_) with their 84% confidence intervals (equivalent to statistical tests at the α < 0.05 level). Different letters indicate significant differences between sampling sites. The Botanical Garden and Puvočiai village have the highest and most similar species richness compared to Brinkiškės village and Verkiai Regional Park.

**Figure 6:**
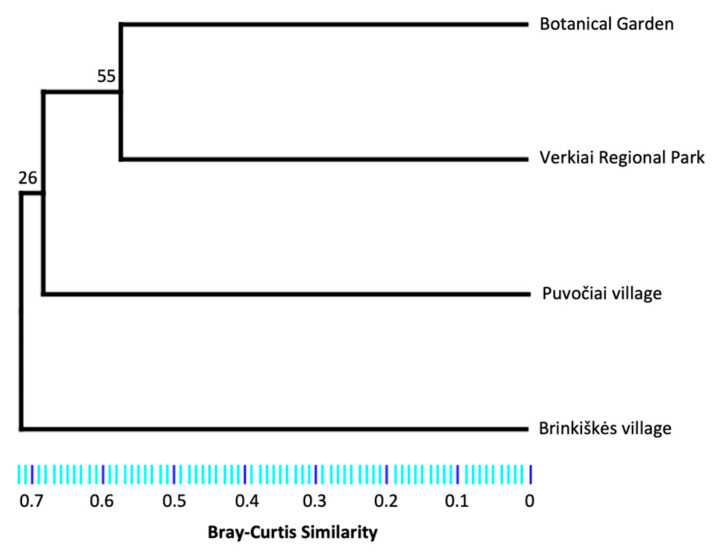
Dendrogram of cluster analysis showing the similarity in *Culicoides* species by sampling site, estimated with the Bray–Curtis index. Numbers on nodes are the probability of group occurrence in 100 bootstrap replications.

*Culicoides* species richness from the Botanical Garden and Puvočiai village were significantly higher compared to Brinkiškės village and Verkiai Regional Park (Figure 5). The most similar sites in terms of *Culicoides* species composition were the Botanical Garden and the Verkiai Regional Park (Figure 6). Puvočiai village clustered separately from these two sites, and the most contrasting site is Brinkiškės village (Figure 6). 

Haemosporidian parasite DNA was detected in 154 biting midges (7.9%) belonging to *C. pictipennis* (95 individuals), *C. kibunensis* (42), *C. festivipennis* (six), *C. segnis* (five), *C. obsoletus* complex (four), *C. impunctatus* (one), and *C. reconditus* (one) (Table 1). In total, ten species and 23 lineages of haemosporidian parasites were detected. In addition, nine *Haemoproteus* species and 20 lineages were identified, including four newly molecularly described lineages (CULKIB04, CULKIB05, CULPIC01, and CULPIC02). Additionally, one *Plasmodium* species and three lineages were identified, with two new lineages molecularly described (CULFES01 and CULOBS01). Notably, ten of the recovered parasite lineages are still not identified down to the species level (Table 1). Eight samples showed signs of co-infections by different *Haemoproteus* lineages.

The highest number of *Culicoides* parous females, PCR-positive individuals, and the highest prevalence of parasite lineages was detected at the Botanical Garden, followed by the Verkiai Regional Park, Puvočiai, and Brinkiškės villages (Table 3 and Table 4). Of all the recovered lineages, 16 were detected in only one study site, 12 being exclusively found at the Botanical Garden. Only *H. asymmetricus* (TUPHI01) and *H. minutus* (TURDUS2) were detected in all localities, being the lineages with the highest prevalences (Table 4).

**Figure 7:**
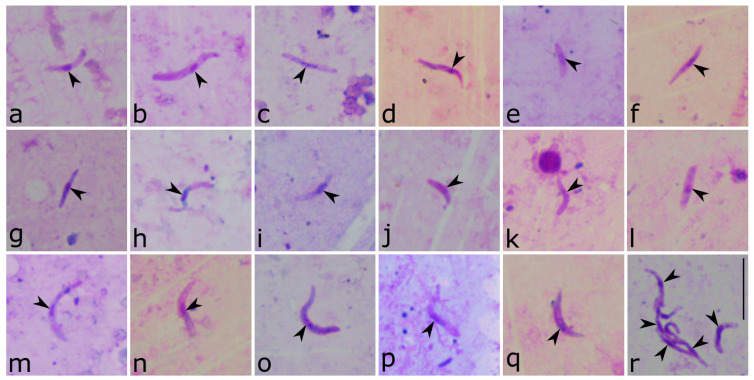
Sporozoites found in the salivary gland preparations of *Culicoides festivipennis* (**a**), *C. kibunensis* (**b**,**f**,**h**,**j**,**l**,**o**), *C. pictipennis* (**c**,**d**,**g**,**i**,**k**,**m**,**p**,**r**), *C. reconditus* (**e**), and *C. segnis* (**n**,**q**). These sporozoites belong to *Haemoproteus belopolskyi* cytochrome *b* lineage HIICT1 (**a**), *H. homominutus* CUKI1 (**b**), *Haemoproteus* sp. CULPIC02 (**c**), *H. homogeneae* SYAT16 (**d**), *H. magnus* ROFI1 (**e**), *H. parabelopolskyi* SYAT01 (**f**,**g**,**r**) and SYAT02 (**h**,**i**), *Haemoproteus* sp. SYAT13 (**j**,**k**), *H. asymmetricus* TUPHI01 (**l**–**n**), and *H. minutus* TURDUS2 (**o**–**q**). Arrowhead indicates sporozoite nucleus. Methanol fixed and Giemsa-stained. Scale bar 10 µm.

Microscopic analysis of salivary gland preparations from the PCR-positive samples showed the presence of sporozoites in 45 (29.2%) of them, being 21 from *C. pictipennis*, 19 from *C. kibunensis*, two *C. festivipennis*, two *C. segnis*, and one from *C. reconditus* (Table 1). This is the first time that *C. reconditus* has been confirmed as a natural vector of *Haemoproteus* parasites, and, in the present study, it was infected by *H. magnus* ROFI1 (Figure 7e). Additionally, this is also the first case that *C. kibunensis* is confirmed as a competent vector of *H. homominutus* CUKI1 (Figure 7b), *H. parabelopolskyi* SYAT01 (Figure 7f) and SYAT02 (Figure 7h), and *Haemoproteus* sp. SYAT13 (Figure 7j). *Culicoides pictipennis* was also confirmed as a competent vector of *H. parabelopolskyi* SYAT01 (Figure 7g), *Haemoproteus* sp. SYAT13 (Figure 7k), *H. homogeneae* SYAT16 (Figure 7d), *H. asymmetricus* TUPHI01 (Figure 6m), and *H. minutus* TURDUS2 (Figure 7p). *Culicoides segnis* had sporozoites of *H. asymmetricus* TUPHI01 (Figure 7n) and *H. minutus* TURDUS2 (Figure 7q), while *C. festivipennis* had sporozoites of *H. belopolskyi* HIICT1 (Figure 7a). Sporozoites of *H. homominutus* CUKI1, *H. magnus* ROFI1, *H. homogeneae* SYAT16, *H. parabelopolskyi* SYAT01, and *Haemoproteus* sp. CULPIC02 (Figure 7c) and SYAT13 (Figure 7j,k) are being reported for the first time. In one of the samples, many sporozoites were identified, sometimes all in the same field (Figure 7r). One of the samples presenting a co-infection was positive by microscopy (Table 1). All insects that were PCR-positive for *Plasmodium* DNA were negative on microscopy.

The sporozoites from different *Haemoproteus* parasites and lineages are morphologically very similar to each other (Figure 7), being readily recognized in the salivary gland preparations by an elongated shape, with an average length that usually exceeds 10 µm, with their ends being approximately equally pointed, and a more or less centrally located nucleus [7]. Despite that, some slight morphological differences and similarities were noted, i.e., sporozoites of *H. homominutus* CUKI1 (Figure 7b), *H. asymmetricus* TUPHI01 (Figure 7l–n), and *H. minutus* TURDUS2 (Figure 7o–q) seem to have a bigger length and width than the other ones. On the other hand, sporozoites of *H. parabelopolskyi* SYAT01 and SYAT02 (Figure 7f–i) seem to be thinner and shorter than the later ones. Due to the differences in the number of sporozoites on positive salivary gland preparations, their morphometry could not be compared statistically, and it could not be confirmed if this morphological feature can have any taxonomical importance. For the same reason, the comparison between sporozoites from the same *Haemoproteus* lineage found in different *Culicoides* species could not be carried out.

## 4. Discussion

These findings will increase the current knowledge about the host–parasite–vector system for *Haemoproteus* transmission in nature. *Culicoides reconditus* was confirmed as a natural vector of *Haemoproteus* parasites, sporozoites of six *Haemoproteus* lineages were reported for the first time, and 12 new interactions between *Haemoproteus* lineages and *Culicoides* species were identified. The Botanical Garden was the location with the highest abundance of *Haemoproteus* parasite lineages and *Culicoides* species, compared to the other study sites. This might be mainly due to the higher availability of different microhabitats, which include areas of deciduous and coniferous trees, various bushes, naturally occurring and planted plants, presence of ponds and small rivers that give moisture to the soil, dead leaves, and soil rich in organic matter, that would provide breeding sites for the *Culicoides* biting midges. 

All eleven species of *Culicoides* collected and analyzed in the present study had been previously reported in Lithuania [48]. However, it is interesting to mention that a previous study conducted in the same localities reported *C. pictipennis* as one of the least abundant species [11], while, in the present study, it was the biting midge species with the highest abundance (Table 1). This might be due to the differences in the trapping dates. In the previous study, the trapping started at the end of June, and in the present study at the beginning of the same month (C. Chagas personal communication), when these insects are present at higher densities. Yet, fluctuations in densities of insect populations are also a possibility, especially in temperate zones, where these changes are more pronounced and highly dependent on climatic conditions [49,50,51,52,53].

Some *Culicoides* species can be considered as rare in Lithuania, such as *C. fagineus*, *C. albicans*, *C. fascipennis*, *C. newsteadi*, *C. chiopterus*, and *C. deltus* [10,12,13,14]. None of these species were collected in the present study. Other species that are usually collected in small numbers are *C. reconditus*, *C. circumscriptus*, and *C. pulicaris* [52], and our study corroborates with this (Table 1 and Figure 3). Two other species, *C. pallidicornis* and *C. punctatus*, were more abundant in the Botanical Garden and Puvočiai village, respectively, with only a few or no individuals collected in other localities (Table 3 and Figure 3). For example, *C. punctatus* were more commonly found during May and June in the Curonian Spit, Baltic Sea, Lithuania [12], and our sampling did not happen in May, neither at those localities. The prevalence of *C. impunctatus* can also be mentioned since it is also highly abundant in certain regions of the studied area [54], while in others it is found only in low numbers [11]. Our study corroborates these findings, with the highest number of *C. impunctatus* parous females collected in Puvočiai and Brinkiškės villages (Table 3, Figure 3).

As previously mentioned, the differences in sampling time between studies is a possible explanation for these findings [49,50,51,53]. Additionally, the availability of breeding sites for *Culicoides* can also markedly interfere in this abundance and prevalence [55,56,57,58]. As a group, it is possible to say that they occur in organically rich substrates. However, they have a broad range of habitats, including swamps, tree holes, mangroves, shallow margins of ponds, among others [53,59], and a diversity of breeding sites might also be among the main reasons for the diversity of *Culicoides* species in different locations. This is one of the explanations for the high number of observed and expected *Culicoides* species richness found in the Botanical Garden in comparison to the other study sites, as well as for the differences of species found in each locality (Figure 5). Further, the Botanical Garden was second in Shannon diversity, after Brinkiškės village, meaning that *Culicoides* species abundances are more equally distributed than in the other sites (Table 2). This could suggest that these sites are heterogeneous habitats, both in their availability of breeding sites and feeding resources, contrasting with the Verkiai Regional Park that presented the lowest Shannon diversity. The Verkiai Regional Park, despite being located in a peri-urban area, is an extensive forest area. Therefore, this habitat seems to be more homogeneous in its vegetation and breeding sites for biting midges. 

The Botanical Garden presented the highest number of parous females, as well as detected *Culicoides* species, being the only study site where *C. circumscriptus* was detected, and *C. festivipennis*, *C. kibunensis, C. pallidicornis*, and *C. segnis* were present in higher numbers (Table 3). The low numbers of *C. circumscriptus* could be because the traps were placed closer to the ground level (1.5–2 m from the ground), and these biting midges are more commonly found between 20 and 26 m above the ground [30,60]. 

The prevalence of *Haemoproteus* parasites detected using only molecular tools in *Culicoides* was 7.9% (Table 1), which is similar to other studies conducted in Lithuania that used the same integrative approach [10,12,13,14]. The molecular prevalence of the parasites was higher than in Bulgaria [28,29], but lower than in Spain [26]. However, another study conducted in the Czech Republic showed a higher prevalence in the analyzed pools of *Culicoides* insects, with almost half of them being PCR-positive for *Haemoproteus* parasites [61]. In terms of the diversity of *Haemoproteus* lineages, we recovered 20 different ones while the mentioned studies reported a maximum of 11 lineages in Lithuania [10,11,12,13,14] and nine in Bulgaria [29]. In the opposite direction, the investigated samples in the Czech Republic have the lowest diversity of *Haemoproteus* lineages (five) [61]. In the present study, this might be due to the high diversity of study sites, with different degrees of land use and anthropogenic influence on each of them, which would lead to a higher number of bird species living in each area and, consequently, a high diversity of parasite lineages. Most of the mentioned studies also reported the presence of *Plasmodium* DNA in the analyzed insects. This is not an uncommon finding [10,14,21,26,27,62]. However, *Plasmodium* does not complete its sporogonic development in *Culicoides* insects [7,63] and their presence simply indicated that they had a blood meal on an infected bird [9,64].

The Botanical Garden was also the location with the highest diversity of *Haemoproteus* lineages, with 12 out of 20 being reported exclusively at this study site (Table 4). Some *Haemoproteus* parasites species are known for having a high host specificity to their vertebrate hosts at the order level [7]. This is the case of *Haemoproteus* sp. SYAT13 and *H. homogeneae* SYAT16 that, until now, were reported only in *Sylvia atricapilla* and *H. homominutus* CUKI1 which was reported to only be infecting *Turdus philomelos* and *Turdus viscivorus* [65] (Supplementary Table S1). This indicates that these bird species were probably more commonly found in the Botanical Garden than in the other study sites. A recent study conducted in Slovakia has shown that the population of *Sylvia atricapilla* was the most abundant species captured in all seasons, except for winter [66]. This might be the case in Lithuania as well, despite the absence of studies in this sense.

In the opposite direction, other *Haemoproteus* lineages and species are reported in a few bird families and even species [67]. This is the case of two parasite lineages that in this study were reported exclusively in the Puvočiai village, *H. majoris* CWT4 and *H. magnus* ROFI1 (Table 4). Combined, both parasite lineages have been reported in 21 different bird species, 11 for each *Haemoproteus* lineage (Supplementary Table S1). Since these parasites can infect a high diversity of bird species, it is difficult to infer from which of these 21 species the female *Culicoides* biting midge got this infection. *Haemoproteus* sp. CIRCUM05 was found only in the Brinkiškės village, and this parasite lineage was reported only in Corvidae (Supplementary Table S1). Birds from this family are highly successful in cities and areas with anthropogenic influence [68], which is the case of the Brinkiškės village study site. *Haemoproteus syrnii* CULKIB01 is another parasite that has been reported in a few Strigiformes species (Supplementary Table S1) and it was recorded only in the Verkiai Regional Park during our study. Due to the specificity of this parasite lineage to infect hosts from Strigiformes, it is possible to infer that the analyzed *Culicoides* had a blood meal in a bird belonging to this order. Additionally, we can also infer that the Verkiai Regional Park likely has a more suitable environment for this group of birds compared with the other three.

Only *H. asymmetricus* TUPHI01 and *H. minutus* TURDUS2 were found in all study sites and had the highest prevalence (Table 1 and Table 4). They were described in *Turdus philomelos* and *Turdus merula* [7,69], respectively, and are more frequently reported in these bird species, even though they can also be found in several other bird species (Supplementary Table S1). The high diversity of vertebrate hosts might be one of the probable explanations for their high prevalence in this study and for their presence in all four study sites. It is also necessary to mention that *T. philomelos* and *T. merula* are abundant and commonly found in Lithuania during the spring and summer months, with their population showing an increased tendency [70].

This study reports the highest number of co-infections detected by PCR methods in *Culicoides* (double peaks were identified in the electropherograms) compared to previous studies, especially in Lithuania [10,11,12,13,14]. Haemosporidian co-infections are commonly found in naturally infected birds [66,71,72,73,74] and there is the possibility of having more than one parasite lineage developing in the same *Culicoides* individual. Unfortunately, the effects of having more than one parasite developing in the same vector are far from being fully understood [75,76], especially with *Culicoides*, and should be targeted in future studies. One of the individuals with co-infection was also positive for sporozoites; since it is not possible to confirm parasite species based on the sporozoites morphology with the current knowledge, we prefer not to make any inferences. It is also necessary to mention that a high number of parasite lineages described in *Sylvia atricapilla* (SYAT01, SYAT02, SYAT05, SYAT13, and SYAT16) were found in the investigated insects (Table 1). This bird species is well known for the high prevalence of co-infections [66,77], which might also have influenced the high prevalence of co-infections in the studied *Culicoides*.

Remarkably, almost 30% of the PCR-positive *Culicoides* were also positive for *Haemoproteus* sporozoites (Table 3). This was the highest positivity ever reported using this integrative approach (insects’ dissection, microscopy, and molecular-based methods) [10,11,12,13,14]. Some of the salivary gland preparations had just a few sporozoites, while in others, they were seen in high numbers (Figure 7r). This might be due to (i) the recent release of sporozoites by the oocysts or (ii) higher levels of infections of the *Culicoides* females. However, it is difficult to confirm this, since there are no studies related to the life span of *Haemoproteus* sporozoites in the *Culicoides* salivary glands, to the number of sporozoites that can be injected during the female blood meal or even if all these characteristics are species-related or if they differ depending on the vector species.

*Culicoides reconditus* is being reported as PCR-positive and able to support sporogonic development of *Haemoproteus* parasites for the first time. This biting midge species is widespread in Europe [37], but it is usually sampled in small numbers in Lithuania [10,11,14]. However, it was noted to be one of the main *Culicoides* species in blue tit *Cyanistes caeruleus* nest boxes in Germany [78]. Due to their lower numbers, this might not be the main *Haemoproteus* vector in Lithuania, although the present study confirms that it can also be involved in this parasite’s transmission in the country. More studies should be conducted at different locations to understand their feeding preferences, breeding sites, and their role in *Haemoproteus* transmission. 

*Haemoproteus* sporozoites that develop in *Culicoides* are similar to each other [7], but past studies have reported some differences and suggested that morphological analyses could be applied for parasite identification in vectors [79]. Sporozoites of *H. homominutus* CUKI1 (Figure 7b), *H. asymmetricus* TUPHI01 (Figure 7l–n), and *H. minutus* TURDUS2 (Figure 7o–q) seem to have a bigger length and width than the other ones, e.g., *H. parabelopolskyi* (compare Figure 7b with Figure 7g–i). However, without morphometrical and statistical analysis, it is not possible to draw any conclusions. In the present study, the differences in the number of sporozoites in the infected *Culicoides* did not allow such analysis, and future studies addressing this issue should be conducted. Noteworthy, all mentioned lineages are closely related and are known for their fast development into the ookinete stage after the *Culicoides* blood meal, resulting in a structure with small size and no outgrowths, as well as presenting gametocytes with a pale staining cytoplasm [65,69,80,81]. It is worth mentioning that *T. merula* and *T. philomelos* are also commonly found with natural co-infections of haemosporidian parasites not only from different but also from the same genus [82], which can also be an explanation for the high number of co-infections found in our study.

Even though several parasite lineages were detected in the present study, it is still not possible to completely understand the mechanisms that promote and/or limit parasite transmission in nature. However, it is possible to confirm that *C. kibunensis*, *C. pictipennis*, and *C. segnis* are important *Haemoproteus* vectors in Lithuania (Table 1 and Table 3, Figure 3). Not only for being relatively abundant, but also for their ornithophilic habits [83,84,85]. The biology of hosts and vectors should also be considered in future studies targeting this issue. 

## 5. Conclusions

*Culicoides kibunensis, C. pictipennis,* and *C. segnis* are important *Haemoproteus* vectors in Lithuania. *Culicoides reconditus* was reported for the first time to support the complete sporogonic development of *H. magnus cytb* lineage ROFI1. A high number of *Haemoproteus* parasite lineages (23) and a high prevalence of sporozoites (~30% of PCR-positive) in the investigated *Culicoides* females of some species indicated that many parasite species are circulating in Lithuania and that they can be potentially transmitted to birds of different species during their reproductive season, including juveniles. Twelve new associations between *Haemoproteus* lineages and *Culicoides* species were reported. The mechanisms that limit *Haemoproteus* transmission are far from being completely understood, and studies targeting this issue should be encouraged.

## Figures and Tables

**Table 1:** Haemosporidian lineages and associated species isolated from wild *Culicoides* species. PCR-positive samples are indicated for each *Haemoproteus* lineage and biting midge species. The numbers indicated in brackets represent the number of salivary gland preparations positive for sporozoites. *Culicoides circumscriptus* (C), *C. festivipennis* (F), *C. impunctatus* (I), *C. kibunensis* (K), *C. obsoletus* complex (O), *C. pallidicornis* (Pa), *C. pictipennis* (Pi), *C. pulicaris* (Pu), *C. punctatus* (Pn), *C. reconditus* (R), *C. segnis* (S), and *Culicoides* sp. (Sp).

	Pi	O	K	F	I	S	Pa	Pn	R	Sp	Pu	C	Total
*H. asymmetricus* TUPHI01	64 (10)		19 (10)	1		2 (1)							80 (20)
*H. minutus* TURDUS2	15 (2)		10 (5)			1 (1)							26 (8)
*H. parabelopolskyi* SYAT02	4 (3)		1 (1)										5 (4)
*H. belopolskyi* HIICT1	1		1	2 (2)									4 (2)
*H. parabelopolskyi* SYAT01	2 (2)		1 (1)										3 (3)
*Haemoproteus* sp. SYAT13	1 (1)		1 (1)										2 (2)
*H. homominutus* CUKI1			2 (1)										2 (1)
*Haemoproteus* sp. CULPIC02	1 (1)												1 (1)
*H. magnus* ROFI1									1 (1)				1 (1)
*H. homogeneae* SYAT16	1 (1)												1 (1)
*H. majoris* CCF5			2										2
*H. majoris* PARUS1		1				1							2
*Haemoproteus* sp. CCF2			1										1
*Haemoproteus* sp. CULKIB04			1										1
*Haemoproteus* sp. CULKIB05			1										1
*Haemoproteus* sp. HAWF6			1										1
*Haemoproteus* sp. CIRCUM05						1							1
*H. syrnii* CULKIB01	1												1
*Haemoproteus* sp. CULPIC01	1												1
*H. majoris* CWT4		1											1
*Plasmodium* sp. CULFES01				1									1
*Plasmodium* sp. CULOBS01		1											1
*P. vaughani* SYAT05				1									1
Co-infection	4 (1)	1	1	1	1								8
Negative	496	459	409	176	105	49	50	42	4	5	3	1	1799
Total positive	95 (21)	4	42 (19)	6 (2)	1	5 (2)	0	0	1 (1)	0	0	0	154 (45)
Total sampled	591	463	451	182	106	54	50	42	5	5	3	1	1953
Prevalence (%)	15.8	0.9	9.4	3.3	1.0	9.3	0	0	20	0	0	0	7.9

**Table 2:** *Culicoides* sample size (n), number of observed species (D), estimated sample coverage (C), estimated coefficient of variation (CV), and exponential of Shannon entropy (e^H^’) per sampling site with 95% confidence intervals (95% CIs).

Study Site	n	D	C	CV	e^H^’ (95% CI)
Botanical Garden	871	10	0.99	1.39	4.46 (4.20–4.72)
Puvočiai village	490	9	1	1.74	3.35 (2.96–3.73)
Brinkiškės village	107	8	1	0.50	6.93 (6.31–7.54)
Verkiai Regional Park	485	7	0.99	1.64	2.56 (2.36–2.76)

**Table 3:** Ratios of *Haemoproteus* infections according to PCR, and the presence of sporozoites in salivary gland preparations (MIC). Results are presented per *Culicoides* species and study sites: the Botanical Garden (BG), Puvočiai village (P), Brinkiškės village (B), and the Verkiai Regional Park (VRP). Microscopical analyses were conducted only in PCR-positive samples.

	BG		P		VRP		B	
*Culicoides* species	PCR	MIC	PCR	MIC	PCR	MIC	PCR	MIC
*C. pictipennis*	30/197	12/30	8/38	0/8	53/337	7/53	4/19	2/4
*C. obsoletus* complex	0/40	- ^a^	4/314	0/4	0/95	- ^a^	0/14	- ^a^
*C. kibunensis*	37/395	17/37	1/8	0/1	2/32	0/2	2/16	2/2
*C. festivipennis*	3/145	1/3	0/11	- ^a^	0/12	- ^a^	3/14	1/3
*C. impunctatus*	0/7	- ^a^	1/73	0/1	- ^b^	- ^b^	0/27	- ^a^
*C. segnis*	3/34	2/3	0/2	-	1/7	0/1	1/11	0/1
*C. pallidicornis*	0/47	- ^a^	- ^b^	- ^b^	0/1	- ^a^	0/2	- ^a^
*C. punctatus*	0/2	- ^a^	0/35	- ^a^	0/1	- ^a^	0/4	- ^a^
*C. reconditus*	- ^b^	-	1/5	1/1	- ^b^	- ^b^	- ^b^	- ^b^
*Culicoides* sp.	0/3	- ^a^	0/2	- ^a^	- ^b^	- ^b^	- ^b^	- ^b^
*C. pulicaris*	0/1	- ^a^	0/2	- ^a^	- ^b^	- ^b^	- ^b^	- ^b^
*C. circumscriptus*	0/1	- ^a^	- ^b^	- ^b^	- ^b^	- ^b^	- ^b^	- ^b^
Total	73/871	32/73	15/490	1/15	485	7/49	107	5/5

^a^ All insects tested were PCR-negative. ^b^ *Culicoides* species not found at the study site.

**Table 4:** Distribution of parasite lineages according to the locations. The Botanical Garden (BG), Puvočiai village (P), Brinkiškės village (B), and the Verkiai Regional Park (VRP).

Parasite Species and Lineage	BG	P	VRP	B
*Haemoproteus asymmetricus* TUPHI01	37	6	39	4
*H. minutus* TURDUS2	11	3	9	3
*H. parabelopolskyi* SYAT02	2		3	
*H. belopolskyi* HIICT1	3			1
*H. parabelopolskyi* SYAT01	1		2	
*Haemoproteus* sp. SYAT13	2			
*H. homominutus* CUKI1	2			
*Haemoproteus* sp. CULPIC02	1			
*H. magnus* ROFI1		1		
*H. homogeneae* SYAT16	1			
*H. majoris* CCF5	2			
*H. majoris* PARUS1		1	1	
*Haemoproteus* sp. CCF2	1			
*Haemoproteus* sp. CULKIB04	1			
*Haemoproteus* sp. CULKIB05	1			
*Haemoproteus* sp. HAWF6	1			
*Haemoproteus* sp. CIRCUM05				1
*H. syrnii* CULKIB01			1	
*Haemoproteus* sp. CULPIC01	1			
*H. majoris* CWT4		1		
*Plasmodium* sp. CULFES01	1			
*Plasmodium* sp. CULOBS01		1		
*P. vaughani* SYAT05	1			
Co-infection	4	2	1	1
Negative	798	475	429	97
**Total**	**871**	**490**	**485**	**107**

## Data Availability

The data presented in this study are available in the Supplementary Materials, in the GenBank database (https://www.ncbi.nlm.nih.gov/genbank/, accessed on 20 November 2023, accession numbers PP003069-PP003214 and OR995548-OR995567), and in the MalAvi database (http://130.235.244.92/Malavi/, accessed on 20 November 2023). Representative salivary gland preparations are available on request at the Nature Research Centre (accession numbers NS49742-NS49757).

## Supplementary Materials

The following supporting information can be downloaded at: https://www.mdpi.com/article/10.3390/insects15030157/s1, Supplementary Table S1: *Haemoproteus* and *Plasmodium* lineages found in the present study and their vertebrate host vectors. The data were compiled based on the information available in the MalAvi database (http://130.235.244.92/Malavi/, accessed on 20 November 2023).
